# Mechanobiology of Periodontal Ligament Stem Cells in Orthodontic Tooth Movement

**DOI:** 10.1155/2018/6531216

**Published:** 2018-09-17

**Authors:** Huaming Huang, Ruili Yang, Yan-heng Zhou

**Affiliations:** Department of Orthodontics, Peking University School and Hospital of Stomatology, National Engineering Laboratory for Digital and Material Technology of Stomatology, Beijing Key Laboratory of Digital Stomatology, Beijing 100081, China

## Abstract

Periodontal ligament stem cells (PDLSCs) possess self-renewal, multilineage differentiation, and immunomodulatory properties. They play a crucial role in maintaining periodontal homeostasis and also participated in orthodontic tooth movement (OTM). Various studies have applied controlled mechanical stimulation to PDLSCs and investigated the effects of orthodontic force on PDLSCs. Physical stimuli can regulate the proliferation and differentiation of PDLSCs. During the past decade, a variety of studies has demonstrated that applied forces can activate different signaling pathways in PDLSCs, including MAPK, TGF-*β*/Smad, and Wnt/*β*-catenin pathways. Besides, recent advances have highlighted the critical role of orthodontic force in PDLSC fate through mediators, such as IL-11, CTHRC1, miR-21, and H_2_S. This perspective review critically discusses the PDLSC fate to physical force *in vitro* and orthodontic force *in vivo*, as well as the underlying molecular mechanism involved in OTM.

## 1. Introduction

Orthodontic tooth movement (OTM) is induced by mechanical forces and is promoted by the remodeling of periodontal ligament (PDL) and alveolar bone. Under the stimulation of appropriate orthodontic force, the periodontal tissue is reconstructed at the molecular, cellular, and tissue levels [1]. On the compression side, the PDL becomes compressed and disorganized and it results in bone resorption, while the stretching of PDL fibers induces bone deposition on the tension side [2].

Orthodontists successfully correct malocclusion by orthodontic treatment. However, there are still some challenges for them. For example, excessive orthodontic forces or common forces on the teeth of periodontitis patients may cause periodontium damage. What is more, mechanical force-induced orthodontic root resorption and relapse after treatment are still major clinical challenges in orthodontic treatment.

Since 2004, periodontal ligament stem cells (PDLSCs) have been separated and shown to share characteristics with mesenchymal stem cells (MSCs) [3]. Having immunomodulatory function and potential for proliferation and generation of cementum/periodontal ligament-like complex [3, 4], PDLSCs play important roles in periodontal homeostasis. And they are likely sensitive to mechanical loading and play critical roles in periodontal and osseous remodeling during OTM. A better understanding of the mechanical response of PDLSCs and the cellular signaling pathway involved may help in solving the challenges in orthodontic treatment.

The role of PDLSCs responding to orthodontic force during the tooth movement *in vivo* has been confirmed [5]. *In vitro*, recent advances have also clarified that the applied mechanical force, including tension, compression, and vibration, can significantly regulate the proliferation and differentiation of cultured PDLSCs. The data is summarized in Table 1 to compare expediently the response of PDLSCs to different mechanical force and summarize the effect that the duration, frequency, and magnitude have on cellular fate, which may help in understanding why low force is critical in achieving physical bone remodeling in orthodontic treatment. In this review, we include the studies focusing on PDLSCs instead of periodontal ligament cells (PDLCs) isolated according to cell culture methods and identification of the multipotency of stem cells [5–15]. In order to find out the role of PDLSCs in OTM, we expound the response of PDLSCs to orthodontic force *in vivo* and mechanical force *in vitro* and also summarized the critical related mechanosensors and mechanism pathways.

## 2. The Effects of Orthodontic Force on PDLSCs during the Tooth Movement *In Vivo*

Orthodontic tooth movement is induced by the constant application of orthodontic force, which is promoted by PDL and alveolar bone remodeling. The force exerted on the teeth is transferred to the alveolar bone through PDL. It is generally agreed that compression causes bone resorption, which is regarded as the rate-limiting step, while tension leads to bone formation [1]. Several studies have explored the role of PDLSCs during OTM *in vivo*.

Zhang and colleagues established an OTM rat model and used PDGFR*α* and nestin to track the response of rat PDLSCs (rPDLSCs) *in vivo* [5]. They found that after 3 days of orthodontic treatment, the number of PDGFR*α* or nestin-positive cells increased on both of the compression and of the tension sides and then dropped after 7 days, suggesting that rPDLSCs may be reactivated on both sides during orthodontic force treatment.

Besides, rPDLSCs play a role in PDL recovery and orthodontic relapse process. After the orthodontic force is removed, PDL can return to its original structure and then orthodontic relapse occurs [16]. Feng and colleagues demonstrated that upon orthodontic force, the density of PDL collagen, as an important component of extracellular matrix in PDL, reduced on the compression side and recovered after force was removed for 5 days [6]. Correspondingly, the expression of type I collagen (Col-I) in rPDLSCs was declined with orthodontic force applied and recovered after force removal.

## 3. The Molecules Transmitting the Orthodontic Force to PDLSCs *In Vivo*

During OTM, a variety of molecules help in transducing the force signals into PDLSCs, such as interleukin- (IL-) 11, collagen triple helix repeat containing 1 (CTHRC1), microRNA-21 (miR-21), and hydrogen sulfide (H_2_S).

IL-11 is produced by a variety of PDLCs, including fibroblasts, osteoclasts, and osteoblasts [17]. It is known to be associated in the regulation of osteoclasts and osteoblasts as well as bone remodeling [18]. After OTM, it was observed that IL-11 increased in the rat PDL [19]. Correspondingly, IL-11 could stimulate osteoblastic markers and cementoblast-specific markers, increase the expression of bone sialoprotein, and promote the proliferation of human PDLSCs (hPDLSCs). These indicated that force-induced IL-11 was secreted by PDLCs and it was good for hPDLSCs to differentiate into osteoblasts or cementoblasts [19].

CTHRC1 plays an important role in the differentiation of bone MSCs [20]. Wang and colleagues showed that the expression of CTHRC1 was upregulated in PDLCs during OTM and the osteogenic differentiation of hPDLSCs could be promoted by the overexpression of CTHRC1 *in vitro*, demonstrating that osteogenic differentiation of hPDLSCs was positively regulated by CTHRC1 during OTM [21].

MicroRNAs, small noncoding RNAs, could be mechanically sensitive and act as key posttranscriptional regulators in osteogenic differentiation of PDLSCs following orthodontic force. Microarray data indicated that 53 microRNAs in hPDLSCs were differentially expressed after tension [22], including hsa-miR-21, which was found to be involved in tension-induced osteogenesis of hPDLSCs *in vitro* [23]. Using wild and miR-21-deficient (miR-21^−/−^) OTM model of rats, Chen and colleagues observed that miR-21 improved force-induced alveolar bone formation on the tension side during OTM in wild rats, which was suppressed in miR-21^−/−^ rats [24]. Furthermore, for the first time, hPDLSCs were collected from donors with or without OTM. The expression of miR-21 was upregulated with promoted osteogenesis in the cultured hPDLSCs following OTM, which was blocked by the inhibition of miR-21. Interestingly, even after the removal of orthodontic force, the increase in cultured hPDLSC osteogenesis was preserved, suggesting an epigenetic effect.

H_2_S is a gaseous transmitter that has recently been associated with the function of MSCs and bone metabolism [25]. It has been elucidated that the production of force-induced H_2_S in hPDLSCs modulated the accumulation of macrophage and osteoclastic and osteogenic activities in the alveolar bone through regulation of the secretion of monocyte chemoattractant protein-1 and the receptor activator of the nuclear factor-*κ*B ligand/osteoprotegerin (RANKL/OPG) system and then controlled the process of OTM [26]. Using a mouse OTM model, Liu and colleagues observed that orthodontic force application elevated the production of H_2_S and upregulated cystathionine *β*-synthase (CBS) in PDL. Moreover, most of the expression of CBS was colocalized with CD90 (an MSC marker). Correspondingly, the secretion of compression-induced H_2_S in the supernatant of cultured hPDLSCs was associated with the CBS expression change in hPDLSCs. Furthermore, blocking the production of endogenous H_2_S suppressed the orthodontic force-induced macrophage accumulation and osteoblasts on the tension side and osteoclasts on the compression side, with a decrease in the distance of OTM. The study showed that PDLSCs generated H_2_S to transduce and respond to force stimulation.

## 4. The Effects of Mechanical Force on the Function of PDLSCs *In Vitro*

To study the mechanobiology of PDLSCs during OTM, lots of studies applied tension and compression mimicking the force on both sides of teeth during OTM to cultured PDLSCs *in vitro*. And other types of mechanical stimulation also have been reported, including vibration, ultrasound, and microgravity (Table 1).

### 4.1. Tension

It has been reported that tension was important for the regulation of ligament tissue remodeling [27]. In one study, the tension of 3000 *μ*strain (nearly 0.3%) at 0.5 Hz was applied to hPDLSCs for 3 h, 6 h, 12 h, and 24 h [7]. The expression of runt-related transcription factor-2, osterix, and Satb2 was significantly upregulated in hPDLSCs in a time-dependent manner, indicating an early response to osteogenic orientation. Other groups also confirmed that tension induced late-stage osteogenic transcription markers, such as osteocalcin [8].

PDLSCs transform tension into different cell behaviors, depending on the manner in which tension is applied, including mechanical devices, magnitude, duration, and frequency. When Pelaez and colleagues applied 5% tension at 0.5 Hz for 2 h to hPDLSCs, they exhibited induced expression of cardiac-specific transcription factors [9]. Another study found that the dome-shaped tension promoted PDLSCs to differentiate into keratocytes, which had synergistic effects with induction medium [10].

To investigate the effect of tension on the function of PDLSCs to regulate osteoclastogenesis, PDLSCs were applied to static mechanical strain (SMS) with a range of magnitudes from 6% to 14% at 0.1 Hz for 12 h [11]. When the strain was less than 12%, the osteoclastic genes (RANKL) showed no significant differences. However, when the strain was higher than 12% in PDLSCs, the levels of osteoclastic genes were obviously increased [11].

What is more, PDLSCs obtained from periodontitis patients (PPDLSCs) and healthy donors (HPDLSCs) respond differently to tension. When PPDLSCs and HPDLSCs were exposed to SMS with a range of magnitudes from 6% to 14% at 0.1 Hz for 12 h, Liu and colleagues observed that different magnitudes of SMS exerted distinct effects on HPDLSCs and PPDLSCs [11]. For HPDLSCs, the best SMS value for the balance between osteogenesis and osteoclastogenesis was 12%, while the optimal force for PPDLSCs was 8%. Excessive SMS would damage the function of both HPDLSCs and PPDLSCs. Furthermore, compared to HPDLSCs, PPDLSCs showed decreased osteogenic activity, activated osteoclastogenesis, and greater secretion of inflammatory cytokines, which indicated that PPDLSCs are more sensitive and less tolerant to SMS.

The study done by Liu and colleagues also showed that without the addition of osteogenic supplements, the best SMS value for optimizing proliferation was 12% for HPDLSCs [11]. In contrast, upon application of tension at the same level, HPDLSCs cultured in osteogenic media exhibited decrease in proliferation [8].

In conclusion, tension can regulate the differentiation and proliferation of PDLSCs *in vitro*. For PDLSCs from healthy donors, tension with a magnitude of 12% could increase osteogenic differentiation and proliferation of PDLSCs and tension above 12% would upregulate the function of PDLSCs to regulate osteoclast differentiation. Thus, the best SMS value for balance osteogenesis and osteoclastogenesis was 12%, while the optimal force for PPDLSCs was 8%. This could explain why lighter force should be used during OTM, especially for periodontitis patients. In addition, similar force magnitudes could lead to different differentiation paths, which may be due to the different culture medium and mechanical devices [9–11].

### 4.2. Compression

Static compression is commonly used *in vitro* to mimic the force on the compressive side during OTM. It has been reported to result in the altered morphology and the differentiation of PDLSCs.

It was found that the morphology and osteogenic gene expression of hPDLSCs responded to compression and would recover after force withdrawal [6]. Upon application of compression at 1g/cm^2^ for 12 h and 24 h, hPDLSCs obtained significantly denser actin distribution and elongated morphology. In addition, the expression of collagen matrix and osteogenic marker (Col-I) in hPDLSCs was suppressed, resulting in a broken and disorganized pattern of PDL collagen. However, both the morphology and decreased gene expression recovered after force withdrawal.

To simulate the compression to hPDLSCs during the OTM process, Zhang and colleagues used a hydraulic-controlled cellular strain element [5]. The compression on cells was produced by continuous compression of 2% CO_2_ and 95% N_2_. Exposed to 100 kPa static hydraulic pressures, hPDLSCs exhibited increased osteogenic differentiation after applying force for 1 h, while osteogenic differentiation of hPDLSCs remained or reduced after 12 h. On the contrary, the ratio of RANKL/OPG was decreased after 1 h, while upregulated after 12 h, which meant that the oclastogenesis was inhibited after 1 h but promoted after 12 h.

In general, short-term compression (applying force for 1 h) could promote osteogenic differentiation of PDLSCs, while long-term compression (applying force for 12 h or longer) inhibits osteogenesis and promotes osteoclastogenesis by increasing the RANKL/OPG ratio. These may be one of the reasons why compression causes bone resorption and acts as the rate-limiting step.

### 4.3. Vibration

Applying low-magnitude, high-frequency (LMHF) vibration at 0.3g with a frequency of 10–180 Hz for 30 mins to hPDLSCs, Zhang and colleagues confirmed that there was a tendency to reduce the proliferation and upregulate the osteogenic differentiation of hPDLSCs as the frequency of stimulation increased, which peaked at 50 Hz [12]. In another study, they processed the LMHF vibration at 50 Hz with a magnitude of 0.05–0.9g for hPDLSCs and found that vibration was most beneficial for the osteogenesis of hPDLSCs at 50 Hz with 0.3g magnitude [13]. In conclusion, LMHF vibration decreases the proliferation and promotes the osteogenesis of hPDLSCs in frequency-dependent and magnitude-dependent manners and the optimal frequency and magnitude are 50 Hz and 0.3g, respectively [12, 13].

### 4.4. Others

With a frequency in the low-megahertz range (1–3 MHz), low-intensity pulsed ultrasound (LIPUS) is widely used as a safe and minimally noninvasive application for regeneration and tissue repair [28]. Indeed, a study showed that the application of LIPUS accelerated the healing of periodontal tissue *in vivo* [29]. In another study, treated with 1 MHz LIPUS for 5/20 minutes, rPDLSCs exhibited increased proliferation, indicating that LIPUS can promote the expansion of PDLSCs [14].

Three-dimensional (3D) dynamic simulation of microgravity induced by a rotary system also had effects on hPDLSCs, and it would benefit their proliferation and osteogenic differentiation [15]. Simultaneously, the morphology of hPDLSCs was changed from a triangular or spindle shape to a sphere shape body.

## 5. Molecules Linking Applied Force with the Fate of PDLSCs *In Vitro*

As mentioned above, applied force is a key regulator for the fate of PDLSCs. Understanding the cellular signaling pathway involved in the mechanical response of PDLSCs is essential for the future improvements in orthodontic therapy.

### 5.1. Mechanosensors

It is not yet clear how cells recognize the mechanical force and convert it into cellular signals. Various mechanosensors have been proposed, including the cytoskeleton, membrane channels, primary cilia, focal adhesions, and gap junctions [30]. However, just the cytoskeleton and Piezo channel have been studied in PDLSCs.

The cytoskeleton consists mainly of actin, microtubules, and intermediate filaments. As the continuous structure between cell membrane and chromosome, the cytoskeleton supplies a structural framework for cells. Mechanical force may change cytoskeletal tension by the change of cell shape [31]. After LMHF mechanical stimulation, the F-actin fibers in hPDLSCs became clearer and thicker [13]. Furthermore, the level of cytoskeletal remodeling influenced the mechanically driven osteogenic commitment of PDLSCs, which was correlated to the magnitude of the applied vibration stimulus, suggesting that the cytoskeleton was involved in transmitting mechanical forces into cells [13].

Piezo is a mechanosensitive membrane ion channel. Mechanical stress can regulate the differentiation of stem cell in the midgut of mature Drosophila via the tension-activated Piezo channel [32]. Gao and colleagues also showed that the Piezo channel was likely to play an important role in conducting ultrasound-related signals in dental pulp stem cells [33]. In the same study, however, they observed that the Piezo channel was not associated with LIPUS-stimulated rPDLSC proliferation.

### 5.2. Mechanotransduction Pathways

Multiple pathways mediate the response of PDLSCs to mechanical stimulation. The functions of the mitogen-activated protein kinase (MAPK), transforming growth factor-*β* (TGF-*β*), and Wnt/*β*-catenin pathways are discussed in more detail below.

Force-induced proliferation of rPDLSCs can involve the activation of MAPK signaling pathways. When LIPUS was applied to rPDLSCs, the c-Jun N-terminal kinases (JNK) MAPK signaling was immediately activated and p38 MAPK kinase was activated 4 hours after the exposure [14]. At the same time, the inhibitions of JNK and p38 reduced LIPUS-associated proliferation of rPDLSCs.

TGF-*β* is a critical molecule in extracellular matrix remodeling and tissue homeostasis [34]. When hPDLSCs are exposed to compression, the expressions of TGF-*β*1 and TGF-*β*3 in hPDLSCs were suppressed after force treatment and recovered following force withdrawal, which was consistent with the alteration of Col-I expression and PDL collagen [6]. Besides, blocking the TGF-*β*/Smad pathway could inhibit the recovery of Col-I expression and PDL collagen during early orthodontic relapse [6].

The Wnt/*β*-catenin pathway plays important roles in the regulation of osteogenic differentiation of PDLSCs. The canonical pathway is involved in translocating *β*-catenin into the nucleus. Zhang and colleagues found that compression activated the Wnt/*β*-catenin pathway and increased the levels of glycogen synthase kinase 3 *β* (GSK-3*β*), active-*β*-catenin proteins, and phospho-GSK-3*β* in hPDLSCs [5]. Furthermore, Dickkopf-related protein 1, the inhibitor of the canonical Wnt pathway, could block the osteogenic differentiation and reinstate the ratio of RANKL/OPG following force treatment.

## 6. Conclusion and Perspective

**Figure 1:**
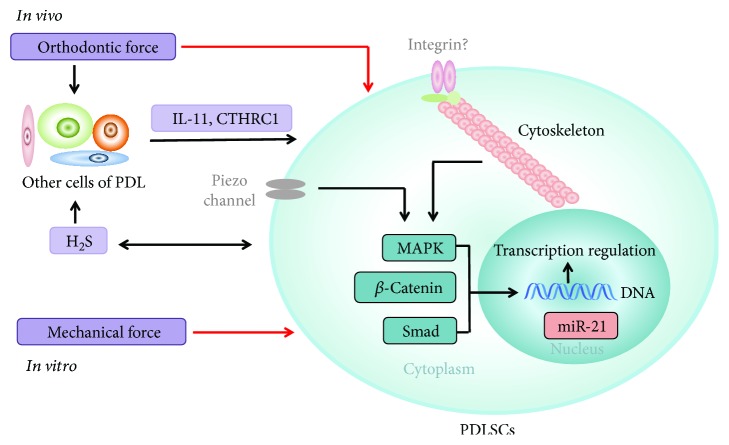
The mechanical forces could act directly on PDLSCs both *in vitro* and *in vivo* (red arrows), while *in vivo*, they may influence PDLSCs indirectly through the factors secreted by other PDLCs (black arrows). A variety of molecules is involved in regulating the fate of PDLSCs after the application of force. IL-11, CTHRC1, miR-21, and H_2_S have been shown to help in transducing orthodontic force signals to PDLSCs. The cytoskeleton and MAPK, TGF-*β*/Smad, and Wnt/*β*-catenin pathways also play important roles in it.

PDLSCs play an important role in OTM. rPDLSCs may be reactivated on both sides during orthodontic force treatment and participate in the orthodontic relapse process. *In vitro*, both tension and compression can regulate osteogenic differentiation and proliferation of PDLSCs, which is consistent with *in vivo* experiments. Under the optimal magnitude, which is 12% for HPDLSCs and 8% for PPDLSCs, tension could increase osteogenic differentiation and proliferation of PDLSCs. And it would upregulate the function of PDLSCs to regulate osteoclast differentiation when the magnitude is above optimal. Short-term compression could promote osteogenic differentiation of PDLSCs, while long-term compression inhibits osteogenesis and promotes osteoclastogenesis. Besides, LMHF vibration decreases the proliferation and promotes the osteogenesis of hPDLSCs and the optimal frequency and magnitude are 50 Hz and 0.3g, respectively. A variety of mechanosensors and pathways are involved in it, including cytoskeleton, MAPK signaling, TGF-*β*/Smad, and Wnt/*β*-catenin pathways. IL-11, CTHRC1, MiR-21, and H_2_S have also been shown to be important to transduce orthodontic force signals into PDLSCs *in vivo* (Figure 1).

This article reviewing the relationship between PDLSCs and mechanical force is crucial to understand the healing process during orthodontic tooth movement, which may help orthodontics to control the orthodontic procedure more effectively. As mentioned above, excessive force will damage the function of PDLSCs, so lighter force should be used during OTM, especially for periodontitis patients. Due to their proliferation and differentiation potential, PDLSCs could potentially be used for the reconstruction of periodontal tissues, which might accelerate the procedure of orthodontic treatment.

However, there are several important issues that still need to be investigated. Firstly, it needs to seed PDLSCs on a culture plate of proper rigidity or three-dimension scaffolds *in vitro* to find the optimal method to mimic the nature environment during OTM. In addition, investigating the role of extracellular matrix and other potential molecules such as integrin and Erk1/2 MAPK, which have been found to be mechanotransduction in PDLCs [35], will help in figuring out how the force is transduced into PDLSCs. Furthermore, whether the effects of PDLSCs on peripheral blood mononuclear cell and T cell are regulated by orthodontic force and why the PPDLSCs respond differently to force need to be addressed. Finally, investigating the function of PDLSCs in orthodontic root resorption and orthodontic relapse will contribute to understanding OTM and control the major clinical challenges in orthodontic treatment.

## Figures and Tables

**Table 1:** Effects of applied force induced the function of PDLSCs.

Force types	Cell source	Culture	Mechanical devices and parameter	Discoveries
*Tension*				
[7]	Premolars from donors aged 12–18	In alpha minimum essential medium (*α*-MEM); on pure plates	Self-made four-point bending system; cyclic tension; 0.5 Hz, 0.3%, 3 h, 6 h, 12 h, and 24 h	Increased osteogenic markers
[8]	Premolars from donors aged 12–24	In osteoinductive medium; on collagen I-bonded 6-well plates	Flexcell FX-4000T Tension Plus System; cyclic tension, 0.1 Hz, 12%, 6 h, 12 h, and 24 h	Increased osteogenic markers, decreased proliferation
[9]	Third molars	In Dulbecco's modified Eagle medium (DMEM); on collagen I-coated membranes	Custom-built bioreactor system; cyclic tension, 0.5 Hz, 5%, 2 h	Increased markers of cardiomyogenesis
[10]	Third molars	In *α*-MEM; on collagen I-bonded Bioflex 6-well plates	Flexcell Tension System; dome-shaped equibiaxial static mechanics	Increased keratocyte markers
[11]	Premolars and third molars from healthy and chronic periodontitis patients	In *α*-MEM; on collagen I-bonded Bioflex 6-well plates	Flexcell FX-4000 T Tension Plus System; static tension, 0.1 Hz, 6%, 8%, 10%, 12%, and 14%, 12 h	Optimal magnitude in promoting proliferation and osteogenic activity is 12% for HPDLSCs and 8% for PPDLSCs
*Compression*				
[6]	Third molars from donors aged 19–29	In medium containing 6 mM of Ca^2+^	A layer of glass cover and metal weights; static compression, 1g/cm^2^, cultured for 12 h and 24 h after force withdrawal	Altered cell morphology and repressed collagen expression, which both recovered after force withdrawal
[5]	Healthy teeth	In *α*-MEM; on pure plates	Hydraulic pressure-controlled cellular strain unit; 1000g/cm^2^, 1 h and 12 h	Increased and reduced osteogenic markers after 1 h and 12; reduced and upregulated ratios of RANKL and OPG after 1 h and 12 h, respectively
*Vibration*				
[12]	Premolar from donors aged 12–16	In *α*-MEM; on parallel six-well plates	GJX-5 vibration sensor; 10–180 Hz, 0.3g, 30mins/24 h	Decreased proliferation and increased osteogenic markers in a frequency-dependent manner, with significant peaks at 50 Hz
[13]	Premolar from donors aged 12–16	In *α*-MEM; on parallel six-well plates	GJX-5 vibration sensor; 50 Hz, 0.05g to 0.9g, 30mins/24 h	Decreased proliferation and increased osteogenic markers in magnitude-dependent manners, with significant peaks at 0.3g; no obvious senescent cells
Ultrasound [14]	Six-week-old male Wistar Han rats	In *α*-MEM; on 6-well plates	A DuoSon therapeutic ultrasound device; 1 MHz, 5 or 20 mins	Increased proliferation
Microgravity [15]	Premolars and third molars	In DMEM; on Cytodex 3 microcarriers	Rotating bioreactor; 15 rpm, 24 h	Alterations of morphology, increased proliferation, and osteogenic differentiation
